# The regression time of ocular surface squamous neoplasia using topical interferon alfa-2b does not depend on the initial tumor size

**DOI:** 10.5935/0004-2749.20220018

**Published:** 2025-08-21

**Authors:** Magí Vilaltella, Valentín Huerva

**Affiliations:** 1 Department of Ophthalmology, University Hospital Arnau de Vilanova, Lleida, Spain; 2 Surgery Department, Faculty of Medicine, University of Lleida, Lleida, Spain

**Keywords:** Conjunctival neoplasm, Corneal disease/drug therapy, Carcinoma, Squamous cell, Interferon alpha-2/therapeutic use, Neoplasia da túnica conjuntiva, Doença da córnea/ tratamento farmacológico, Carcinoma de célula escamosa, Interferon alfa-2/ uso terapêutico

## Abstract

**Purpose:**

The aim of this study was to determine if the initial tumor size correlates with the time to regression after topical interferon alfa-2b (1 million IU/mL) therapy in the treatment of ocular surface squamous neoplasia.

**Methods:**

A retrospective study was performed in 15 patients clinically diagnosed as having ocular surface squamous neoplasia and treated with topical interferon alfa-2b (1 million IU/mL, four times a day). All the cases of ocular surface squamous neoplasia included in the study had corneo-limbal involvement. The initial extension of the ocular surface squamous neoplasia was measured in square millimeters using the program ImageJ (LOCI, University of Wisconsin, Madison, USA) on images taken from the eyes of each patient immediately before the beginning of the treatment. The time until tumor resolution was measured for each case.

**Results:**

Complete tumor resolution was achieved in all the cases, with a mean initial tumor extension of 26.71 mm^2^ (standard deviation ± 17.21 mm^2^) and a mean time until resolution of 77 days (standard deviation ± 32 days). An increased tumor volume after 15 days of treatment was observed in 2 patients, which completely resolved. No significant correlation was found between the time to resolution and the initial tumor extension measured in square millimeters (Spearman test, p=0.347).

**Conclusions:**

Our study suggests that the duration of topical interferon alfa-2b treatment required does not depend on the initial tumor size of the ocular surface squamous neoplasia usually found in clinical practice.

## Abstract (pt)

**Objetivo:**

O objetivo deste estudo é determinar se o tamanho inicial do tumor se correlaciona com o tempo de regressão após terapia tópica com interferon alfa- 2b (1 milhão de UI/ml) no tratamento da neoplasia escamosa da superfície ocular.

**Métodos:**

Foi realizado um estudo retrospectivo em 15 doentes clinicamente diagnosticados com neoplasia escamosa da superfície ocular e tratados com interferon tópico alfa- 2b (1 milhão de UI/ml, quatro vezes ao dia). Todos os casos de neoplasia escamosa da superfície ocular incluídos no estudo apresentaram envolvimento da córnea e do limbo. A extensão inicial da neoplasia escamosa da superfície ocular foi medida em milímetros quadrados usando o programa ImageJ (LOCI, University of Wisconsin, Madison, EUA) em imagens feitas dos olhos de cada paciente imediatamente antes do início do tratamento. O tempo até a resolução do tumor foi observado em cada caso.

**Resultados:**

A resolução tumoral completa foi alcançada em todos os casos, com média da extensão inicial de 26,71 mm^2^ (desvio padrão ± 17,21 mm^2^) e tempo médio até a resolução de 77 dias (desvio padrão ± 32 dias). Um aumento do volume tumoral após 15 dias de tratamento foi observado em 2 pacientes com resolução completa do tumor. Não houve correlação significativa entre o tempo até a resolução e a extensão inicial do tumor medida em milímetros quadrados (teste de Spearman, p=0,347). **Conclusões** Nosso estudo sugere que a duração do tratamento necessário com interferon alfa- 2b tópico não depende do tamanho inicial do tumor de neoplasia escamosa da superfície ocular geralmente encontrada na prática clínica.

## INTRODUCTION

Squamous neoplasias, which are neoplastic epithelial lesions of the conjunctiva and cornea that include dysplasic and invasive carcinomas, are currently called ocular surface squamous neoplasia (OSSN). Histologically, the term OSSN includes squamous neoplasias contained in the basement membrane, conjunctival intraepithelial neoplasia (CIN), and those that invade the stroma (invasive squamous cell carcinoma). Clinically, the term *conjunctival intraepithelial neoplasia* has fallen out of favor in preference to the more general term *ocular surface squamous neoplasia* because it is not possible to determine on clinical examination whether stromal invasion has occurred.

The incidence of OSSN is low in high-latitude countries in the northern hemisphere, but the incidence increases in equatorial areas^(1-3)^. OSSN occurs predominantly in males in the seventh decade of life and is predominantly localized to the corneal limbus^(1,2)^.

The management of OSSN involves the use of topical chemotherapeutic agents such as mitomycin C (MMC), 5-fluorouracil (5-FU), and interferon alfa-2b (IFN)^(1,4)^. Topical IFN alfa-2b is useful as adjuvant or primary treatment in the management of OSSN^(1,4-7)^. The gold standard for OSSN treatment is still surgery^(1)^; however, surgery without adjuvant treatment is associated with high recurrence rates. Surgery may also maim the ocular surface in extensive cases that invade the limbus^(6-8)^. Topical IFN alfa-2b is an ideal medication for the treatment of OSSN because it is relatively nontoxic^(4,9-11)^. IFN alfa-2b is a highly purified protein that binds to cell receptors and triggers the synthesis of effector proteins that inhibit cell growth and cell differentiation in an antineoplastic action, activate immunocompetent cells, and regulate oncogenes. IFN also has an antiviral effect that involves a natural defense mechanism^(9-11)^. Many previous studies focused on its efficacy and safety for OSSN treatment^(1,2,4-8,12-15)^. The drawback of this treatment is its long treatment duration of 8-16 weeks^(1,5,6,8,9)^. The treatment time may increase if the topical administration of IFN alfa-2b is continued 1 or 2 months after resolution, with a mean total therapy duration of 4 months^(5)^. Furthermore, whether the treatment duration depends on the initial extent of the tumor or on the tumor volume is unclear.

On the basis of these considerations, the aim of this study was to determine if the initial tumor size, in smalland medium-sized OSSNs, correlates with the time to regression after topical IFN therapy.

## METHODS

**Figure 1:**
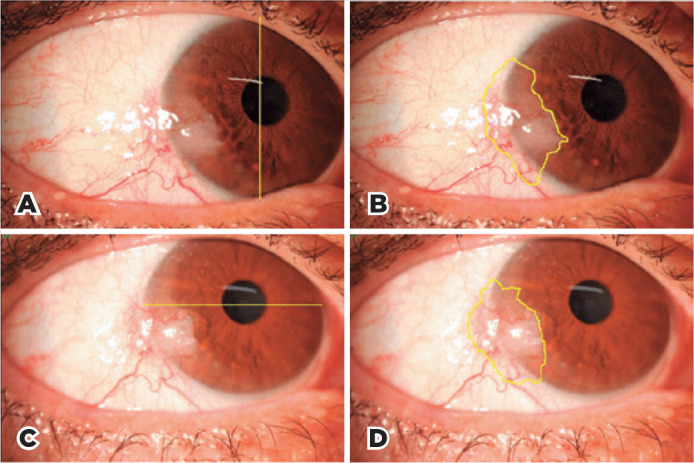
Photographs of Patient 11 on day 1 (A, B) and day 15 (C, D) after the beginning of the treatment. A. The yellow line indicates the vertical white-to-white distance, which is given a value of 10.63 mm to set a scale for measuring the tumor area on the following image. B. The yellow line outlines the shape of the tumor, and the area is automatically calculated by the program Image J using the scale established on image A. C. As the horizontal diameter is more visible in this case, the yellow line from W-W on the horizontal plane is traced and given a value of 11.46 mm to set a scale for measuring the tumor area on the following image. D. The yellow line outlines the shape of the tumor, and the area is automatically calculated by the program Image J using the scale established on image C

A retrospective study was performed in 15 consecutive patients who were diagnosed as having corneo-limbal OSSN and treated with topical IFN alfa-2b (1 million IU/mL) four times a day, from 2009 to 2018, at the Ocular Surface Unit of the Ophthalmology Department of the University Hospital Arnau de Vilanova de Lleida. Treatment was continued until the lesion was fully resolved. The diagnosis and resolution of the lesion were both clinically determined. Resolution was defined as the total disappearance of the OSSN based on clinical observation. No further diagnostic methods were performed. Informed consent was obtained from each patient prior to treatment. The study was conducted in accordance with the ethical standards and adhered to the tenets of the Declaration of Helsinki. The patients were fully informed about the examinations, treatments, and possible interventions. Written consent was obtained from all the subjects. All the tumors were of stage T1 or T2 according to the 8th edition of the Classification of the American Joint Committee on Cancer^(9,16)^. All the OSSN cases included in the study had corneo-limbal involvement. The initial extension of OSSN was measured in square millimeters using the program ImageJ (LOCI, University of Wisconsin). To set the scale, the horizontal or vertical white-to-white (W-W) distance was used in each case at our convenience, with a vertical W-W distance of 10.63 mm and a horizontal W-W distance of 11.46 mm as references, according to previous studies on mean corneal diameter in adult population^(17)^. After setting the scale, the tumor perimeter was outlined using the computer mouse, and the area was calculated by the program (Figure 1). This task was carried out in all the cases by the same blinded examiner. The measurements were determined from photographs taken at the beginning of the treatment and in the consecutive visits. Only photographs in which >75% of the corneal diameter was visible were included in the study to correctly estimate the scale and, therefore, the actual extension of the tumor. The purpose of this method was to obtain the maximum objective measure of the tumor extension before and in the consecutive visits after treatment. Tumor extension was measured at the beginning of the treatment (day 0) and after 15 days to record any increase in tumor volume.

The patients were followed up every 15 days using photographs to verify the treatment response, establish if complete tumor resolution had occurred, determine the time required for tumor resolution, and record any increase in tumor volume. After observing complete resolution of the tumor, IFN alfa-2b drops were stopped immediately, and a follow-up control was made 6 and 12 months after.

Statistical analyses were performed using the SPSS ver. 24.0 software for Windows (SPSS Inc., Chicago, IL, USA). Nonparametric correlation analyses were performed. The Spearman correlation was used to assess the correlation between the time to resolution and the initial size of the tumor.

## RESULTS

**Figure 2:**
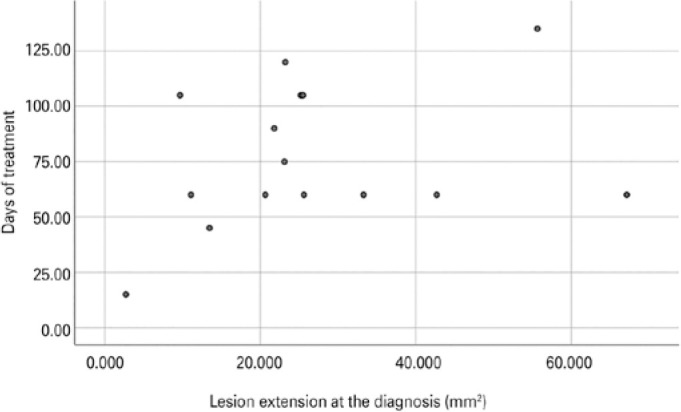
Scatter plot of the correlation between lesion extension at diagnosis and period of treatment until resolution, showing no correlation between the two variables.Spearman test, p=0.347(The scatter plot that appears in this file has not enough image quality.

Fifteen patients with corneo-limbal OSSN involvement were included in the study. The median patient age was 70.8 years. Eleven patients (73%) were men, and 4 (26%) were women (Table 1). All the patients had Caucasian (Spanish) ancestry. The mean tumor extension was 26.71 mm^2^ (median, 23.23 mm^2^; standard deviation ± 17.21 mm^2^). Complete regression was observed in all the cases after a mean time of 77 days of treatment (median, 60 days; standard deviation ± 32 days; Table 2). When comparing tumor extensions on day 0 of treatment and after 15 days, as measurements were taken in square millimeters, small differences may not be significant, so we established the line of significant increase or decrease in tumor extension at 2 mm^2^. Taking this into consideration, during the first 2 weeks, 2 cases showed an increase in tumor extension, 8 showed a decrease in tumor extension, and 5 had the same extension (Tables 3 and 4). When the time to tumor regression was evaluated on the basis of the initial size on the ocular surface, no statistically significant correlation was observed (Spearman test: p=0.347; Figure 2). Nevertheless, the smallest tumor appeared to regress faster than the others, and the second largest tumor regressed more slowly than any other tumor (Figure 2). No recurrences were observed in any case among the follow-up controls at 6 and 12 months after complete tumor regression.

**Table 1:** Demographics of the patients

Patient	Sex	Age (years)
1	F	82
2	F	81
3	M	83
4	M	77
5	F	53
6	M	52
7	M	62
8	M	69
9	M	75
10	M	67
11	F	63
12	M	81
13	M	65
14	M	78
15	M	74
	Male percentage: 73%	Mean: 70.8
	Female percentage: 26%	Median: 74 Standard deviation (SD): 10.21

**Table 2:** Initial tumor extension and time to resolution

Patient	Day 1 (tumor extension in mm^2^)	Time to resolution (days)
1	33.286	60
2	25.604	60
3	21.776	90
4	23.096	75
5	2.695	15
6	11.076	60
7	25.220	105
8	9.661	105
9	13.445	45
10	55.666	135
11	20.652	60
12	23.230	120
13	67.151	60
14	25.490	105
15	42.687	60
	Mean: 26.71	Mean: 77
	Median: 23.23	Median: 60
	Standard deviation: ± 17.21	Standard deviation: ± 32.00

**Table 3:** Initial tumor extension and tumor extension after 15 days of treatment

Patient	Day 1 (tumor extension in mm^2^)	Day 15 (tumor extension in mm^2^)
1	33.286	30.586
2	25.604	27.406
3	21.776	23.941
4	23.096	25.123
5	2.695	0
6	11.076	8.395
7	25.220	27.900
8	9.661	7.290
9	13.445	5.954
10	55.666	48.997
11	20.652	22.018
12	23.230	13.689
13	67.151	66.356
14	25.490	21.130
15	42.687	43.398
	Mean: 26.71	Mean: 24.81
	Median: 23.23	Median: 23.941
	Standard deviation: ± 17.21	Standard deviation: ± 17.72

**Table 4:** Tumor extension changes after 15 days of treatment

	Increase	Decrease	Same
Number of cases	2	8	5
% of cases	13%	53%	33%

## DISCUSSION

Topical IFN alfa-2b (1 million IU/mL) is effective for achieving complete tumor regression as a primary treatment for OSSN^(4-9,12-15,18-20)^. Tumor regression occurred in 100% of the cases in the present study. The regression rate oscillates between 70% and 100% in other series of cases^(1,5,12-15,19,21)^. Differences in stability among the IFN eye drops could explain this variability^(8)^, although no studies have investigated IFN eye drop stability and the degree of preservation of the principal active component. Moreover, topical IFN alfa-2b eye drops have been reported to be effective in well-delimited and extensive cases^(7,9,18-20)^. No significant differences in the rate of complete regression of OSSN have been reported according to dose, which can range from 1 to 3 million IU/mL^(21)^. The 1-million IU/mL dose is used most commonly.

The main advantage of topical IFN is that mutilating surgery can be avoided in cases with lesions that extensively affect the limbus. However, the treatment duration can be long^(1,4,5)^. The mean treatment duration in our study was 77 days, which agrees with certain studies that reported durations of 75-180 days^(1,4,5,12-15,18-20)^. However, other studies reported longer durations^(9,22)^. Treatment duration has not been associated to tumor extension. The possibility that a larger lesion may require a longer time to resolve has been suggested previously^(12)^. It is logical to assume that a widely extensive tumor will require a longer treatment period, although this remains to be proven. The aim of the present study was to corroborate this hypothesis.

This study is the first to prove the primary hypothesis on the relationship between the initial tumor size and time to complete regression for OSSN treated with IFN alfa-2b. In previous reports, the tumor size of OSSN has been measured using limbal clock hours affected, the basal diameter in mm or a combination of the two major diameters^(9,12,15,19,22)^. In another study, tumor size was measured by calculating the surface area using geometric formulas for areas depending on the shape of the lesion^(9)^. In our work, we calculated the surface area by outlining exactly the shape of the tumor on an image of the eye, achieving a much more objective, exact, and reproducible measurement. The treatment response in our study was monitored every 15 days, which allowed us to estimate very approximately the exact time to tumor regression in each case.

For these reasons, the present study shows, with more accurate measurements, that by using the same standard dose in all patients, the treatment duration did not depend on the initial tumor extension. A previous study that included 23 cases of OSSN treated with IFN alfa-2b also reported that tumor extension did not correlate with the time taken to respond to topical IFN^(9)^. However, a different method of measurement was used, and in our study, the frequency of follow-up visits for monitoring the treatment response was higher in comparison with that in the other work (15 days *vs.* 3-6 months). For these reasons, the results of both studies are not comparable.

Kim et al. observed a median time of 5 months to complete tumor resolution in 7 cases of giant OSSN when treating them with topical IFN^(22)^, whereas we observed a median time of 60 days in a sample of OSSN cases that did not include any giant tumor. Our study included tumor sizes that are typically found in clinical practice, not including giant OSSNs, which may have a different nature, so the results are not comparable.

Although some authors suggest the administration of IFN alfa-2b in subconjunctival injections^(5,6)^, we decided to perform treatment with topical drops only owing to its simplicity and comfort for the patient.

An increase in tumor volume at the beginning of treatment was previously reported^(18)^. In addition, spontaneous intratumoral bleeding after 3 weeks of topical therapy was reported in one patient^(15)^. In the present study, the extension increase reported in two patients resolved completely. Tumor extension increase may be secondary to a local immune reaction, which does not require the suspension of treatment. Although some authors decide to taper the frequency of IFN alfa-2b after clinical resolution^(6)^, in our study, we stopped the treatment immediately after clinical resolution and did not observe any recurrence during the follow-ups at 6 and 12 months after complete tumor regression.

This study has some limitations. First, it was difficult to recruit a large number of patients with OSSN who were treated with IFN alfa-2b because of the low prevalence of these tumors in high-latitude countries (0.13-1.9 cases per 100,000 population)^(1,2,23)^. Second, the study only assessed tumor extension, and not the thickness of the lesion. Thickness measurements with optical coherence tomography (OCT) might be interesting for further studies. Third, diagnosis and resolution were clinically determined; no further methods such as impression cytology or staining were performed.

In conclusion, our study suggests that the duration of topical IFN alfa-2b treatment required does not depend on the initial tumor size of smallto medium-sized OSSNs.

## References

[1] Huerva V, Ascaso FJ., Srivastava S (2012). Intraepithelial neoplasia.

[2] Ramberg I, Heegaard S, Prause JU, Sjö NC, Toft PB. (2015). Squamous cell dysplasia and carcinoma of the conjunctiva. A
nationwide, retrospective, epidemiological study of Danish
patients. Acta Ophthalmol.

[3] Gichuhi S, Sagoo MS, Weiss HA, Burton MJ. (2013). Epidemiology of ocular surface squamous neoplasia in
Africa. Trop Med Int Health.

[4] Huerva V, Mangues I. (2008). Treatment of conjunctival squamous neoplasias wit interferon
alpha 2ab. J Fr Ophtalmol.

[5] Sayed-Ahmed IO, Palioura S, Galor A, Karp CL. (2017). Diagnosis and medical management of ocular surface squamous
neoplasia. Expert Rev Ophthalmol.

[6] Nanji AA, Moon CS, Galor A, Sein J, Oellers P, Karp CL. (2014). Surgical versus medical treatment of ocular surface squamous
neoplasia: a comparison of recurrences and complications. Ophthalmology.

[7] Siedlecki AN, Tapp S, Tosteson AN, Larson RJ, Karp CL, Lietman T (2016). Surgery versus interferon alpha-2b treatment strategies for
ocular surface squamous neoplasia: a literature-based decision
analysis. Cornea.

[8] Huerva V., Nanji Re: (2014). Surgical versus medical treatment of ocular surface squamous
neoplasia: a comparison of recurrences and complications. Ophthalmology.

[9] Shah SU, Kaliki S, Kim HJ, Lally SE, Shields JA, Shields CL. (2012). Topical interferon alfa-2b for management of ocular surface
squamous neoplasia in 23 cases: outcomes based on American Joint Committee
on Cancer classification. Arch Ophthalmol.

[10] Smith M, Trousdale MD, Rao NA, Robin JB. (1989). Lack of toxicity of a topical recombinant interferon
alpha. Cornea.

[11] Baron S, Tyring SK, Fleischmann Jr. WR, Coppenhaver DH, Niesel DW, Kilimpel GR (1991). The interferons. Mechanisms of action and clinical
applications. JAMA.

[12] Karp CL, Moore JK, Rosa RH. (2001). Treatment of conjunctival and corneal intraepithelial neoplasia
with topical interferon alpha-2b. Ophthalmology.

[13] Schechter BA, Koreishi AF, Karp CL, Feuer W. (2008). Long-term follow-up of conjunctival and corneal intraepithelial
neoplasia treated with topical interferon alfa-2b. Ophthalmology.

[14] Sturges A, Butt AL, Lai JE, Chodosh J. (2008). Topical interferon or surgical excision for the management of
primary ocular surface squamous neoplasia. Ophthalmology.

[15] Kusumesh R, Ambastha A, Sinha B, Kumar R. (2015). Topical Interferon a-2b as a single therapy for primary ocular
surface squamous neoplasia. Asia Pac J Ophthalmol.

[16] McGowan HD. (2009). Squamous neoplasia of the conjunctiva: the new TNM classification
by the American Joint Committee on Cancer (AJCC). Ophthalmology Rounds.

[17] Khng C, Osher RH. (2008). Evaluation of the relationship between corneal diameter and lens
diameter. J Cataract Refract Surg.

[18] Huerva V, Sánchez MC, Mangues I. (2007). Tumor-volume increase at beginning of primary treatment with
topical interferon Alpha 2-b in a case of conjunctiva-cornea intraepithelial
neoplasia. J Ocul Pharmacol Ther.

[19] Schechter BA, Schrier A, Nagler RS, Smith EF, Velasquez GE. (2002). Regression of presumed primary conjunctival and corneal
intraepithelial neoplasia with topical interferon alpha-2b. Cornea.

[20] Huerva V, Mateo AJ, Mangues I, Jurjo C. (2006). Short-term mitomycin C followed by long-term interferon alpha 2
beta for conjunctiva-cornea intraepithelial neoplasia. Cornea.

[21] Galor A, Karp CL, Chhabra S, Barnes S, Alfonso EC. (2010). Topical interferon alpha 2b eye-drops for treatment of ocular
surface squamous neoplasia: a dose comparison study. Br J Ophthalmol.

[22] Kim HJ, Shields CL, Shah SU, Kaliki S, Lally SE. (2012). Giant ocular surface squamous neoplasia managed with interferon
alpha-2b as immunotherapy or immunoreduction. Ophthalmology.

[23] Giaconi JA, Karp CL. (2003). Current treatment options for conjunctival and corneal
intraepithelial neoplasia. Ocul Surf.

